# Health Care Utilisation in a Cohort of Patients with Primary and Secondary Antibody Deficiency in the United Kingdom

**DOI:** 10.1007/s10875-024-01809-3

**Published:** 2024-09-27

**Authors:** Benjamin Dimbleby, Will Greenway, Siobhan O. Burns, Alex G. Richter, Adrian M. Shields

**Affiliations:** 1 Clinical Immunology Service, School of Infection, Inflammation and Immunology, University of Birmingham, Birmingham, UK; 2 Department of Immunology, Royal Free London NHS Foundation Trust, London, UK; 3 Institute of Immunity and Transplantation, University College London, London, UK; 4 Department of Clinical Immunology, University Hospitals Birmingham NHS Foundation Trust, Birmingham, UK

**Keywords:** Inborn Errors of Immunity, Primary Immunodeficiency, Secondary Immunodeficiency, Healthcare Resource use, Rare Disease

## Abstract

**Introduction:**

This study investigates the frequency of hospital attendances, emergency care attendances and geographical influences on service interaction in cohorts of patients with primary and secondary antibody deficiency, to inform future service planning and delivery.

**Methods:**

The COVID-19 in Antibody Deficiency (COV-AD) study was a United Kingdom study that enrolled 525 participants between April 2021 and September 2022. Data on health care utilisation was extracted from a screening cohort of participants at one participating site (Birmingham, UK). Hospital attendance (i.e. all outpatient and inpatient care episodes, including hospital-based IVIG treatment) and emergency care attendance patterns were analysed. Geographical differences in travel times to hospitals and associated costs were considered for all participants at all recruiting sites.

**Results:**

Individuals with antibody deficiency had a median of 7 hospital attendances per year. A diagnosis of secondary antibody deficiency, and antibody deficiency severe enough to require treatment with immunoglobulin replacement were associated with an increased frequency of hospital attendance. 12.7% of the cohort attended the Emergency Department at least once in the preceding twelve months. Individuals with secondary antibody deficiency were at greater risk of requiring emergency care over the preceding one-year and five-year periods. Individuals receiving subcutaneous immunoglobulin lived further from their local immunology centre and were more likely to engage with the COV-AD research study remotely, via dried blood spots sampling.

**Conclusion:**

This study highlights the utilisation of emergency and secondary care usage amongst patient with immunodeficiency and may inform service adaptation and development to better accommodate patient needs and circumstances.

## Introduction

Individuals with primary and secondary antibody deficiencies are prone to infectious and non-infectious complications of their underlying disease and require long-term follow up from Clinical Immunologists and other specialists. In the United Kingdom, this care is coordinated through a network of thirty Clinical Immunology centres based across the country.

At present, there is no data describing how patients with primary and secondary antibody deficiency utilise secondary and tertiary care services. The COVID-19 pandemic significantly changed the way care was delivered to these cohorts [1]. To mitigate the risks posed by SARS-CoV-2, patients “shielded” and remote care, including telemedicine and subcutaneous home immunoglobulin therapy, was expanded, minimising the need for hospital attendances [1–4].

Although these changes have been made, the optimum way of delivering long-term care and minimising the burden of treatment to patients with antibody deficiency is unclear. UK consensus opinion recommends routine clinical review and phlebotomy every six months for all patients receiving immunoglobulin replacement therapy [5]. However, if the number of patients with antibody deficiency requiring immunoglobulin replacement continues to increase [6], traditional, face-to-face clinical services may have to adapt to accommodate growing demand. Furthermore, qualitative studies have outlined the benefits and potential impacts of remote care on individuals and their families [7].

Data describing how patients with antibody deficiency utilise secondary and tertiary care services are necessary to understand how to plan or reform service delivery. In this study, we leveraged hospital attendance data from four hospitals in the West Midlands region of the United Kingdom to understand the burden of planned and unplanned hospital attendances in a large cohort of individuals with antibody deficiency. We went on to utilise a second data set, from participants enrolled in the national COV-AD study, to understand whether geography affects how individuals interact with Clinical Immunology services and the direct cost implications to them.

## Methodology

### Background

The COVID-19 in antibody deficiency (COV-AD) (REC reference: 21/LO/0162) was a national UK study that enrolled 525 participants with antibody deficiency between April 2021 and September 2022. The principal aim of the study was to examine the immunological response to SARS-CoV-2 infection and vaccination in patients with antibody deficiency [8]. Antibody deficiency was defined as present in any individual receiving immunoglobulin replacement therapy for primary or secondary immunodeficiency, or in any individual with a serum IgG < 4 g/L receiving prophylactic antibiotics for the prevention of recurrent infections. Ten immunology centres recruited participants to the COV-AD study; each participating centre initially screened their complete patient cohorts to identify those meeting the inclusion criteria. These individuals were then approached to participate in the main study. Individuals could participate in COV-AD via face-to-face appointments at their local immunology centre, or remotely via self-sampled dried blood spots returned by post.

### Birmingham COV-AD Screening Cohort

**Fig. 1:**
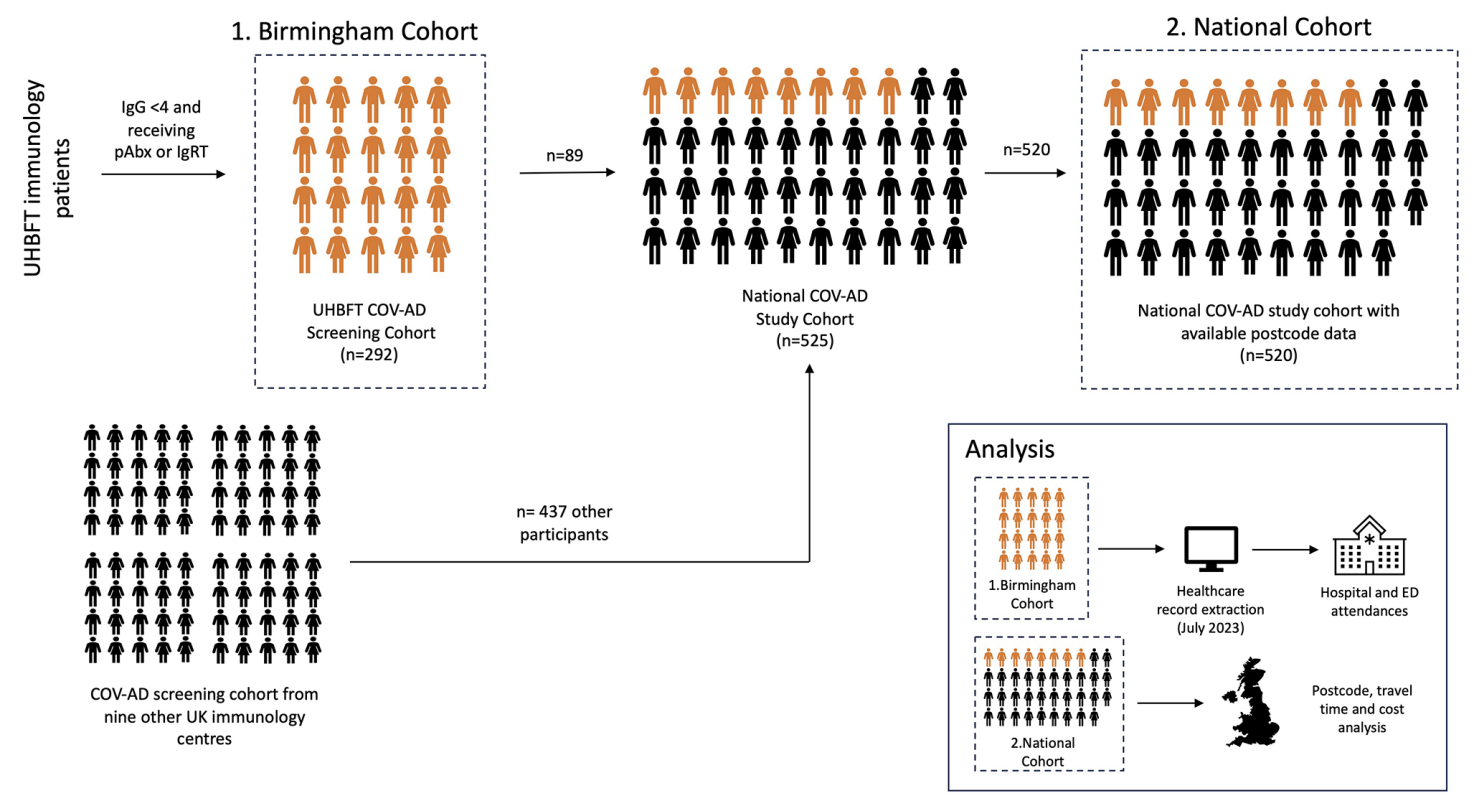
Study design and illustration of cohorts used for data analysis

University Hospitals Birmingham NHS Foundation Trust (UHBFT) administers four hospitals across the West Midlands region providing care for 1.2 million individuals and contributed to the COV-AD study. The UHBFT immunology department identified 292 individuals with antibody deficiency who met the eligibility criteria for COV-AD (Fig. 1**– Birmingham Cohort).** Hospital attendance data over the 5-year period ending July 2023 was gathered from this cohort from the UHBFT patient administration system (PAS) that logs all attendances across all four UHBFT sites. Hospital attendance was defined as any planned or unplanned visit to the hospital recorded on PAS, whether outpatient or inpatient. This includes all attendances for routine outpatient appointments, appointments for immunoglobulin infusions, elective admissions and emergency care. Data on hospital attendances was unavailable for 23 patients (*n* = 23/292, 7.9% of the screened cohort) who had either been lost to long term follow up (*n* = 7) or died (*n* = 16) in the time between COV-AD screening (March 2021) and health care record data extraction for the Birmingham cohort in July 2023.

### COV-AD Cohort

The national COV-AD study cohort (Fig. 1**– National Cohort**) was assembled from participants from UHBFT and 9 other immunology centres across the United Kingdom. COV-AD participants’ residential postcodes, provided at study enrolment, were used to estimate travel distances and costs to their local immunology centre. Postcode data were available for 520 participants. An online search engine (Google Maps) was used to estimate the characteristics of a journey from a participant’s postcode to their named Immunology centre, arriving by 9:00am on 17/11/2023. The distance to hospital by road for each participant was defined as the shortest route, in kilometres. Private transport travel time was determined as the shortest driving route, in minutes. Public transport travel time was determined as the shortest public transport route (using any available forms of public transport), in minutes. Where public transport to Immunology centres was not practically feasible no data was recorded for public transport travel time or cost.

### Private Transport Travel Cost

Estimation of private transport travel costs accounted for travel milage only. At the time of publication, HM Revenue & Customs mileage rates for cars (before 10,000 business miles per year) of £0.45 per mile were applied, as a representative reimbursement, to the shortest round journey by road. Other costs associated with private transport (e.g. congestion charges, ultra-low emission zone charges, motorway tolls and parking charges) were not included.

### Public Transport Travel Cost

For a subset of 72 Birmingham patients for whom public transport had been deemed possible, the costs of public transport were estimated for a 9:00am appointment on 17/11/2023. The cost for an adult for the shortest round trip was calculated using any combination of trains, buses, and trams, and making use of the least expensive or most cost-efficient ticket options (including returns and day tickets, as appropriate).

### Data Analysis

All data analysis were performed using Stata version 17 and GraphPad Prism 10. All continuous outcomes were non-normally distributed and presented as medians with interquartile ranges. Regression analysis used sex (male sex as reference level) and primary vs. secondary antibody deficiency (secondary antibody deficiency as reference level) as binary categorical variable, immunological treatment as a three-level categorical variable (treatment with antibiotic prophylaxis as reference level, IVIG and SCIG as additional categorical levels), with either the number of emergency department attendances or total number of hospital attendances as outcome variables. Statistical comparison of the cost of private transport vs. public transport was performed using a Wilcoxon signed-rank test. Statistical significance was set at *p* < 0.05.

## Results

**Fig. 2:**
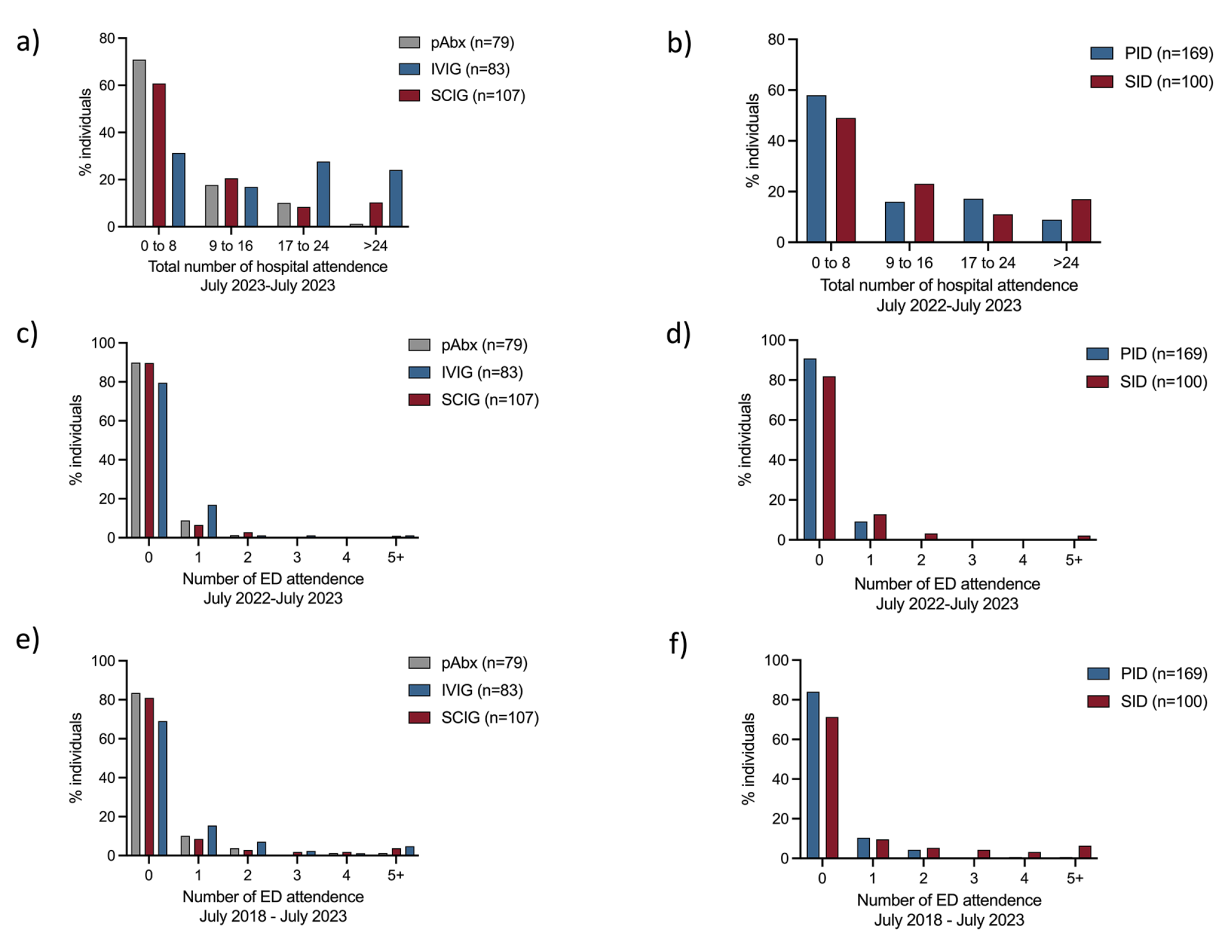
Total hospital and emergency department attendances for Birmingham antibody deficiency cohort (*n* = 292). Total hospital attendances between July 2022 and July 2023 analysed by treatment (**a**) and diagnosis (**b**). Emergency department attendances in individuals with antibody deficiency between July 2022 and July 2023 (**c** & **d**) and July 2018 and July 2023 (**e** & **f**) analysed by treatment and diagnosis

The design of this study and the cohorts used for analysis are summarised in Fig. 1. The demographics of the Birmingham cohort, used to study hospital attendances, are described in Table 1. In this cohort, individuals with antibody deficiency had a median of 7 hospital attendances per annum (IQR 3.0-17.5). Those receiving intravenous immunoglobulin (IVIG) attended hospital significantly more often than those receiving subcutaneous immunoglobulin (SCIG) (median hospital attendance per annum: IVIG: 17 attendances vs. SCIG: 6 attendances, *p* < 0.0001) (Fig. 2a) The proportion of individuals with antibody deficiency who attended hospital more than 24 times for any reason in the year prior to data extraction in July 2023 was 8.8% and 17.0% for primary and secondary antibody deficiency, respectively (Fig. 2b**)**. A multiple regression model adjusted for age, sex, immunodeficiency diagnosis (primary vs. secondary antibody deficiency) and treatment (prophylactic antibiotics, IVIG or SCIG) found a diagnosis of secondary antibody deficiency (compared to primary antibody deficiency), and treatment with either IVIG or SCIG (compared to antibiotic prophylaxis alone) were independently associated with an increased number of hospital attendance in the 12-month period before July 2023 (Table 2). The greatest effect size was seen with treatment with IVIG, commensurate with attendance for hospital-based treatment. However, treatment with SCIG was also associated with increased attendance suggesting an underlying effect related to the severity of underlying antibody deficiency.

**Table 1:** Demographics of Birmingham antibody deficiency cohort (*n* = 292)

**Age** (yr, median, IQR)	62.0 (44.0–73.0)
**Sex** (n, %M)	135 (46.2%)
**Diagnosis (n**,**%)**	
**Primary immunodeficiencies**	
- Common variable immunodeficiency - Combined immunodeficiency - Other primary antibody deficiency - Specific polysaccharide antibody deficiency - X-linked agammaglobulinemia - Other	120 (41.0%) 26 (8.9%) 13 (4.5%) 9 (3.1%) 2 (0.7%) 10 (3.4%)
**Secondary immunodeficiencies**	
- Secondary immunodeficiency	112 (38.4%)
**Immunoglobulin replacement (n**,**%)**	
- Intravenous - Subcutaneous - None	88 (30.1%) 110 (37.7%) 94 (32.2%)

**Table 2:** Linear regression model of determinates of the total number of hospital attendances between July 2022 and July 2023 amongst individuals with antibody deficiency in the Birmingham cohort

Variable	Estimate	Standard error	95% CI (asymptotic)	*P* value
Age	0.0672	0.0513	-0.0339 to 0.168	0.1915
Sex	2.09	1.60	-1.06 to 5.24	0.1922
Secondary antibody deficiency	5.33	1.80	1.79 to 8.86	0.0033
Treatment with IVIG	13.5	2.10	9.37 to 17.7	< 0.0001
Treatment with SCIG	5.26	1.94	1.44 to 9.08	0.0072

Linear regression model uses sex and primary vs. secondary antibody deficiency as binary categorical variables (male sex and secondary antibody deficiency as reference levels), and immunological treatment as a three-level categorical variable with treatment with prophylactic antibiotics as a reference level

12.7% (*n* = 37/292) of the cohort attended the Emergency Department at least once in the preceding twelve months (Fig. 2c **and d**), and 20.9% (*n* = 61/292) had attended the Emergency Department at least once in the past 5 years (Fig. 2e **and f**). Univariate comparison of the percentage of patients with PID or SID accessing emergency care in the preceding year was similar (9.6% PID vs. 17.3% SID, *p* = 0.051), but SID patients were more likely to access emergency care in the preceding five years (15.8% PID vs. 28.7%, *p* = 0.008). A linear regression model found a diagnosis of secondary antibody deficiency (compared to primary antibody deficiency) was independently associated with an increased total number of emergency department attendances in both the 1-year and 5-year periods prior to July 2023 (Tables 3 and 4**)**. Treatment with IVIG, but not SCIG, was also independently associated with an increased number of emergency department attendances in the 5 years prior to July 2023 (Table 4).

**Table 3:** Linear regression model of determinates of the total number of emergency department attendances between July 2022 and July 2023 amongst individuals with antibody deficiency in the Birmingham cohort

Variable	Estimate	Standard error	95% CI (asymptotic)	*P* value
Age	0.00422	0.00256	-0.000823 to 0.00926	0.1006
Sex	0.0379	0.0798	-0.119 to 0.195	0.6348
Secondary antibody deficiency	0.196	0.0896	0.0195 to 0.372	0.0297
Treatment with IVIG	0.183	0.105	-0.0238 to 0.390	0.0826
Treatment with SCIG	0.0906	0.0968	-0.100 to 0.281	0.3503

Linear regression model uses sex and primary vs. secondary antibody deficiency as binary categorical variables (male sex and secondary antibody deficiency as reference levels), and immunological treatment as a three-level categorical variable with treatment with prophylactic antibiotics as a reference level

**Table 4:** Linear regression model of determinates of the total number of emergency department attendances between July 2018 and July 2023 amongst individuals with antibody deficiency in the Birmingham cohort

Variable	Estimate	Standard error	95% CI (asymptotic)	*P* value
Age	0.00692	0.00567	-0.00424 to 0.0181	0.2233
Sex	0.347	0.177	-0.000911 to 0.695	0.0506
Secondary antibody deficiency	0.710	0.198	0.319 to 1.10	0.0004
Treatment with IVIG	0.787	0.232	0.330 to 1.25	0.0008
Treatment with SCIG	0.366	0.214	-0.0563 to 0.788	0.0891

Linear regression model uses sex and primary vs. secondary antibody deficiency as binary categorical variables (male sex and secondary antibody deficiency as reference levels), and immunological treatment as a three-level categorical variable with treatment with prophylactic antibiotics as a reference level

Over the preceding five years, 48.6% of the Birmingham cohort were reviewed by respiratory medicine, 38.0% by haematology, 27.7% by ear, nose and throat surgery and 21.2% by gastroenterology (Table 5). Individuals with secondary immunodeficiency were more likely to be reviewed in secondary care by haematology (66.1% vs. 19.8%) and rheumatology (22.6% vs. 11.3%), reflecting the underlying aetiology of diseases associated with secondary immunodeficiency.

**Table 5:** Specialist clinics attended by adult patients with antibody deficiency between July 2018 and July 2023 (*n* = 292)

	All patients (*n* = 292)		Primary antibody deficiency (*n* = 177)		Secondary antibody deficiency (*n* = 115)	
Specialty	*N*	%	*N*	%	*N*	%
Respiratory medicine	142	48.6	88	49.7	54	47.0
Heamatology	111	38.0	35	19.8	76	66.1
Ear Nose and Throat	81	27.7	37	20.9	44	38.3
Gastroenterology (excl. hepatology)	62	21.2	47	26.6	15	13.0
Dermatology	57	19.5	30	16.9	27	23.5
Rheumatology	46	15.8	20	11.3	26	22.6

To understand the impact and financial cost to individuals with antibody deficiency of attending hospital appointments, we analysed postcode data from 525 individuals enrolled in the national COV-AD study. The enrolment to this cohort is summarised in Fig. 1 and its characterised described in Table 6. The most common diagnoses amongst participants in the study were common variable immunodeficiency (*n* = 233/525, 44.4%) and secondary immunodeficiency (*n* = 139/525, 26.5%). 93.3% (*n* = 490/525) of all participants were receiving immunoglobulin replacement therapy: 48.8% intravenous therapy, which requires regular visits to their immunology centre every three to four weeks; 44.5% of participants were receiving subcutaneous immunoglobulin replacement therapy which is self-administered at home.

**Table 6:** Demographics of national COV-AD cohort (*n* = 525)

**Age** (yr, median, IQR)	58.0 (43.0–69.0)
**Sex** (n, %M)	221 (42.1%)
**Diagnosis (n**,**%)**	
**Primary immunodeficiencies**	
- Common variable immunodeficiency - Other primary antibody deficiency - Specific polysaccharide antibody deficiency - Combined immunodeficiency - X-linked agammaglobulinemia - Other	233 (44.4%) 69 (13.1%) 26 (4.9%) 23 (4.4%) 21 (4.0%) 14 (2.7%)
**Secondary immunodeficiencies**	
- Secondary immunodeficiency	139 (26.5%)
**Immunoglobulin replacement (n**,**%)**	
- Intravenous - Subcutaneous - None - No data	256 (48.8%) 234 (44.5%) 34 (6.5%) 1 (0.2%)
**Pre treatment IgG** (g/L, median, IQR)	3.2 (1.55–4.50)
**Immunoglobulin replacement dose** (g/kg/m, median, IQR)	0.56 (0.47–0.70)
**Trough IgG** (g/L, median, IQR)	9.4 (8.1–11.0)
**Prophylactic antibiotics (n**,** %)**	273 (52.0%)

99.0% (*n* = 520/525) of participants had postcodes from which travel distances to their local immunology centre could be estimated (Table 7). Participants lived a median of 26.7 km from their local Immunology centre. The median travel time by private transport was 35 min compared to 66 min for public transport; heterogeneity in median travel time using both private and public transport was observed between centres nationwide. The relative cost of private and public transport was estimated for the subset of COV-AD participants (*n* = 72) enrolled at two West Midlands centres (Birmingham Heartlands Hospital and Queen Elizabeth Hospital, Birmingham). Although the median cost of a round trip from a participant’s home to their local immunology centre was significantly cheaper using public transport (median cost public transport £9.65 vs. private transport £15.75 (excluding parking charges), *p* < 0.001), in 18.1% of participants, no public transport services existed that would allow a participant to attend a 9am appointment.

**Table 7:** Time and cost implications for individuals involved in travelling to UK immunology centers

Centre	*N* (%)	Patients enrolled remotely (*n*, %)	Distance to immunology centre (km, median, IQR)	Travel time by car to immunology centre (minutes, median, IQR)	Cost of round trip by car (£, median, IQR)	Travel time by public transport to immunology centre (minutes, median, IQR)
Birmingham Heartlands Hospital	54 (10.4%)	46 (85.2%)	32.0 (15.9–53.8)	35 (24–50)	21.01 (12.01–33.16)	87 (59–103)
Bristol Southmead Hospital	53 (10.2%)	39 (73.5%)	27.2 (9.8–49.4)	30 (18–50)	18.91 (9.19–31.33)	84 (46 − 103)
Leeds St James Hospital	32 (6.2%)	31 (97.8%)	18.7 (10.0–29.3)	23 (16–30)	13.49 (8.58–19.38)	72 (51–79)
Newcastle Royal Victoria Infirmary	44 (8.5%)	44 (100.0%)	28.7 (16.9–55.1)	30 (22–53)	20.07 (13.45–34.83)	65 (47–92)
Oxford John Radcliffe Hospital	14 (2.7%)	9 (64.3%)	68.3 (25.9–76.8)	60 (35–65)	40.91 (17.19–45.63)	102 (40–175)
Derriford Hospital	50 (9.6%)	45 (90.0%)	65.7 (23.0–86.3)	50 (28–65)	39.12 (15.27–50.64)	105 (49 − 146)
Queen Elizabeth Hospital, Birmingham	34 (6.5%)	23 (67.6%)	19.6 (9.8–47.8)	27 (16–45)	16.84 (11.39–32.63)	61 (48–88)
Royal Free Hospital, London	209 (40.2%)	129 (61.7%)	24.6 (11.9–51.5)	40 (28–65)	20.17 (13.06–35.20)	60 (41–82)
Salford Royal, Greater Manchester	11 (2.1%)	11 (100.0%)	23.2 (11.7–64.9)	20 (14–45)	14.96 (8.57–38.27)	76 (66–114)
Royal Stoke University Hospital	19 (3.7%)	2 (10.5%)	11.7 (7.7–26.9)	20 (14–30)	9.77 (7.52–18.23)	56 (48–82)
**Total**	**520**	**379** **(72.8%)**	**26.7** **(11.7–54.3)**	**35** **(21–55)**	**19.64** **(11.29–34.69)**	**66** **(45–96)**

We observed that participants using subcutaneous immunoglobulin tended to live further from their local immunology centre (IVIG 35.3 km vs. 44.7 km, *p* = 0.01) and were more likely to participate in the COV-AD study remotely, using self-sampled dried blood spots as a method to donate samples (*p* = 0.001).

## Discussion

Individuals with antibody deficiency are prone to infectious and non-infectious complications [9], yet there is very little data describing the utilisation of primary, secondary, or emergency care by such cohorts. A study examining Hospital Episode Statistics from England and Wales between 1999 and 2019, found 8.4% of a total of 5.8 million hospital admissions secondary to diseases of the blood, blood-forming organs, and immune system were coded as arising from immunodeficiencies [10]. Other studies have shown hospital admissions in individuals with primary immunodeficiency are most commonly due to infection and demonstrate seasonal patterns [11, 12]. In comparison to the general population, hospitalisation rates following SARS-CoV-2 infection were significantly greater in patients with immunodeficiency both before and after the deployment of vaccination [13].

Our data suggests up to 1 in 7 patients with antibody deficiency access emergency care on an annual basis and individuals with secondary antibody deficiency attend the emergency department more frequently, both in the short-term and long-term. These data should prompt reflection on the reasons why individuals with antibody deficiency access emergency care and the development of strategies to facilitate acute admission avoidance. Improvements in the recognition of secondary antibody deficiency may mitigate this risk. Haematological malignancy and its treatments are a major cause of secondary antibody deficiency, and two-thirds of patients in this cohort were also seen by haematologists. We have previously shown the delay from first significant infection to referral to immunology for individuals with haemato-oncological associated secondary immunodeficiency who required immunoglobulin replacement was 49 months; 24% of individuals had developed bronchiectasis by the time of referral [14]. Furthermore, the initiation of antibiotic prophylaxis or immunoglobulin replacement in this cohort was associated with significant reductions in hospital admissions for the treatment of infections [14]. Closer multi-disciplinary coordination at a strategic and individual patient level, between Clinical Immunology and Haematology teams, may help improve the recognition of secondary antibody deficiency, facilitate earlier treatment and reduce front-door pressure on health-care services.

The data presented here also provide insight into the overall burden of hospital attendance for patients with antibody deficiency. Between 8.8% and 17.0% of the cohort attended hospital more than 24 times during a year, depending on the underlying aetiology of their antibody deficiency. Treatment with IVIG and SCIG were both independently associated with an increased frequency of hospital attendances amongst individuals with antibody deficiency, although the effect size was greater with IVIG. This is indicative that receipt of any immunoglobulin replacement is a surrogate of more significant antibody deficiency, associated with a greater burden of treatment; the larger effect size observed with IVIG is most likely driven by increased attendances for hospital-based immunoglobulin infusions. However, treatment with IVIG, but not SCIG, was also independently associated with increased emergency department attendances in the five years prior to July 2023. This effect was not replicated over a one-year period. The reasons underlying these observations require further investigation; one hypothesis is that individuals receiving IVIG possess additional risk factors (e.g. frailty) predisposing them to requiring emergency care, however, this is speculative.

Subcutaneous immunoglobulin treatment is as efficacious in infection prevention as intravenous immunoglobulin replacement and has been associated with reduced overall healthcare cost [15–17]. Furthermore, SCIG is associated with more stable IgG levels and fewer systemic side effects compared to intravenous therapy. Individuals receiving SCIG live, on average, further away from their local centre. It is plausible that geographical distance from their immunology centre and the associated cost of attending hospital appointments may influence an individuals’ and/or a providers’ decision on the mode of immunoglobulin delivery (intravenous vs. subcutaneous therapy).

Expanding the delivery of SCIG where appropriate may also reduce the number of hospital attendances for individuals who require immunoglobulin replacement. As demand for immunoglobulin continues to grow, driven in part by the expanding field of secondary immunodeficiency [6, 18], strategies to deliver safe, patient-centric, supportive immunological care must be developed. For example, multi-speciality clinics may reduce the number of hospital visits for certain patients and technological developments including telemedicine and the use of self-collected dried blood spots to monitor parameters such as immune cell numbers [19] and immunoglobulin concentrations [20] have the potential to safely facilitate the remote care of patients. The planning of innovative services should also consider the financial costs to individuals with chronic conditions of accessing healthcare services. Although we provide estimates of the direct transport costs of attending hospital these likely underestimate the overall financial impact which may also include lost income due to time off work and challenges in maintaining full-time employment around the burdens of chronic illness.

Our study has several limitations. It is uncertain whether the data derived from the UHBFT cohort is generalisable to other centres within the UK or internationally. However, the Trusts’ four hospitals serve a population of 1.2 million individuals and provide tertiary care across a wide geographical area. This may lead to an underestimation of overall service use as individuals who receive their tertiary Immunology care in Birmingham may attend other hospitals for acute or other specialty care, which would not be captured within these data. Also, this study focuses on adult patients with antibody deficiency; patterns of health care use may be different for children. Further research is also needed on how cohorts with antibody deficiency use primary care services. Finally, the COVID-19 pandemic impacted on the way health care was delivered to individuals with antibody deficiency and this study was not specifically designed to quantify that impact. Our analysis focusing on the one-year period between July 2022 and July 2023, over a year after all UK legal restrictions of social interaction were lifted and public services returned to pre-pandemic activities as far as possible, attempts to mitigate the direct impact of the pandemic on service delivery as far as possible. Analysis focusing on the longer five-year period from July 2018 to July 2023 (i.e. emergency department use and other specialists individuals with antibody deficiency interact with) may well be confounded by the pandemic, but provides a larger data set through which services may be better planned.

In conclusion, we provide insight into health care utilisation by a large cohort of individuals with antibody deficiency including data on the logistics of traveling to hospital appointments. These data may inform future service development within Clinical Immunology and associated specialities, particularly given the growing field of secondary antibody deficiency.

## Data Availability

No datasets were generated or analysed during the current study.
